# Therapeutic effect on Alveolar echinococcosis by targeting EM-Leucine aminopeptidase

**DOI:** 10.3389/fimmu.2022.1027500

**Published:** 2022-10-14

**Authors:** Zhen Zhou, Pei Zhou, Yalin Mu, Lei Wang, Zhenjin Cao, Shizhong Dong, Haihua Bao, Baoliang Yang, Minyuan Xin, Runle Li, Ri-Li Ge, Feng Tang

**Affiliations:** ^1^ Research Center for High Altitude Medicine, Qinghai University, Xining, China; ^2^ Qinghai-Utah Joint Research Key Lab for High Altitude Medicine, Qinghai University, Xining, China; ^3^ Qinghai Provincial Key Laboratory of Plateau Medical Application, Key Laboratory of Ministry of Education, Qinghai University, Xining, China; ^4^ Department of Medical Imaging Center, Qinghai University Affiliated Hospital, Xining, China; ^5^ Department of Pathology, The Second Xiangya Hospital DE Central South University, Changsha, China; ^6^ Department of ENT, Qinghai Red Cross Hospital, Xining, China

**Keywords:** Vaccine, Leucine aminopeptidase, *E. multilocularis*, Ubenimex, immune

## Abstract

Alveolar echinococcosis (AE) is a parasitic disease caused by *E. multilocularis* metacestodes and it is highly prevalent in the northern hemisphere. We have previously found that vaccination with *E. multilocularis* Leucine aminopeptidase (EM-LAP) induced specific immune response and had an inhibiting effect on the parasites. In this study, the therapeutic effect of recombinant EM-LAP (rEM-LAP) on AE was evaluated and verified using Ubenimex, a broad-spectrum inhibitor of LAP. The results reveal that rEM-LAP could inhibit cyst growth and invasion and induce specific immunity response in BALB/c mice infected with *E. multilocularis* protoscoleces. The ultrasonic, MRI, and morphological results show that treatment with rEM-LAP inhibits *E. multilocularis* infection and reduces cyst weight, number, fibrosis and invasion. The same effect is observed for the treatment with Ubenimex by inhibiting LAP activity. The indirect ELISA shows that rEM-LAP could induce specific immunity response and produce high levels of IgG, IgG1, IgG2a, IgM, and IgA, and the serum levels of IFN-γ and IL-4 are significantly increased compared to the control groups, indicating that treatment with rEM-LAP leads to a Th1 and Th2 mixed-type immune response. This study suggests that EM-LAP could be a potential therapeutic target of *E. multilocularis* infection.

## Introduction

Alveolar echinococcosis (AE) is a parasitic disease caused by *E. multilocularis* metacestodes and it is prevalent in the northern hemisphere, especially in some developing countries, such as China, Russia, Kazakhstan and Mongolia. The liver is the most commonly involved organ, but other visceral organs, such as lung, brain and spine, may also be involved. Humans may become infected by ingesting food and water contaminated with *E. multilocularis* eggs excreted in the faeces of definitive hosts. Once ingested, the eggs develop into oncospheres that can penetrate the intestine wall and finally reach the liver, where they develop into *E. multilocularis* protoscoleces and cysts and then spread to adjoining tissues (1). The *E. multilocularis* protoscoleces grow like a malignant tumor in the host and will infect the whole liver within only 5-10 years, and it may develop into a “parasitic cancer” and causes cirrhosis and portal hypertension (2).

Surgery and anti-helminthic drugs are the treatment of choice for AE (3). Some proteins of *E. multilocularis* with high immunogenicity could induce immune responses to prevent infection (4–7). Our previous study has shown that vaccination with dominant epitopes, such as EMY162, Glut1, TSP3, 14-3-3 and EM-LAP, could significantly decrease the number and size of cysts in a mouse model infected with *E. Multilocularis* metacestodes. EM-LAP, a metal peptidase of the M17 family, is a potential therapeutic target of *E. multilocularis*. LAP is widely present in many species, such as *E. multilocularis*, *Plasmodium vivax*, *Blood fluke*, and *Fasciola hepatica*, and it plays an important role in the survival, growth, migration, nourishment, molecular assembly and metabolism of parasites (8–10). LAP could cleave n-terminal residues from proteins and peptides, especially leucine substrates (11), and hydrolyzed amino acids are incorporated into parasite proteins. There is evidence that EM-LAP is involved in the infiltrative growth of parasites in the host (12, 13). To sum up, EM-LAP may be effective in treating *E. multilocularis* infection. Our previous study has demonstrated that recombinant EM-LAP (rEM-LAP) reduced *E. multilocularis* infection by activating specific immune responses and releasing specific immunoglobulins and cytokines. Thus, inhibition of EM-LAP activity has the potential to influence *E. multilocularis* growth and metabolism.

To elucidate the role of EM-LAP in *E. multilocularis protoscoleces*, rEM-LAP protein is used as the therapeutic vaccine and its therapeutic efficiency is verified using the broad-spectrum metal peptidase inhibitor (leucine aminopeptidase inhibitor) Ubenimex in this study (14).

## Materials and methods

### Mouse model and treatment

All animal experiments were performed in compliance with the regulations of the Ministry of Science and Technology of China and approved by the Experimental Committee of Qinghai University (QHDX-2019-09). Six to eight weeks old male BALB/c specific pathogen-free (SPF) mice were purchased from Beijing Spaefer Biotechnology Company (SCXK2019-0010) and fed with sterilized feed and water through a 24 h day-night cycle in an animal biosafety level II (ABSL-2) laboratory in the Research Center for High Altitude Medicine 1F (15).

All mice were intraperitoneally injected with 2000 protoscoleces obtained from the Research Center for High Altitude Medicine Basic Immunology Laboratory for Zoonosis (16). After that, mice were randomized into rEM-LAP group, Model group and three Ubenimex concentration groups (2.5 mg/kg, 5 mg/kg, and 7.5 mg/kg) with 6 mice in each group, and they were sacrificed 60 days later for evaluation of the infection, growth and invasion of *E. multilocularis*.

Ubenimex was purchased from Macklin Bio. (CAS: 58970-76-6) and 9.13 mg of Ubenimex was dissolved in the solution consisting of 1100 ul of DMSO, 4400 ul of PEG300, 50 ul of Tween80, and 4950 ul of normal saline. LAP proteins were obtained by prokaryotic expression and purification. Briefly, the EM-LAP gene and protein sequence was retrieved from NCBI and Uniprot, and then the dominant epitopes were selected and connected to Nde I and Xba I of pCzn1 vector by double enzyme digestion to obtain the prokaryotic expression plasmid pCzn1-rEM-LAP, which was transfected into Escherichia coli BL-21 competent cells. The obtained rEM-LAP protein was stored at -80°C (17).

Treatment was started four weeks after protoscolece inoculation. Mice in the rEM-LAP group were injected with 10 ug/day rEM-LAP protein through the tail vein for 60 consecutive days; mice in the Ubenimex group were intraperitoneally injected with 7.5 mg/kg/day, 5 mg/kg/day, or 2.5 mg/kg/day Ubenimex for 60 days; while mice in the Model group were injected with PBS. Samples used for the comparison of therapeutic efficiency were taken from the same mice.

### Ultrasonography

Mice were anesthetized with 1.5% isoflurane in O_2_ and placed on a specially designed heated bed for measurement of the area of cysts in the epigastrium using Ultrasonography (FUJIFILM Vevo^®^ Ultrasonic imager for small animals) one week before sacrifice.

### Magnetic resonance imaging

MRI was performed using a BioSpec^®^ MRI scanner (BRUKER BioSpec94/30 USR) equipped with a set of 80 mm application-specific radiofrequency mouse body coil for *in vivo* imaging. Mice were anesthetized with 1.5% isoflurane in O_2_ and placed on a specially designed heated bed for measurement of cysts in the liver and adjoining tissues. Physiological signals, such as breath rate, were monitored throughout the experiment (18).

### Masson staining of collagen fibers

To determine the invasion in adjoining tissues and the fibrosis level, collagen fibers were stained by Masson trichrome. Samples were optimal cutting temperature (OCT) embedded, sectioned by microtome, and stained using a Masson Trichrome Stain Kit (Solarbio G1340). Images were captured using a microscope (OLYMPUS MX63L) and the positive areas were identified using ImageJ

### Gomori-aldehyde-fuchsin staining of elastic fibers

OCT-embedded liver cysts were sectioned and stained using a Modified Gomori-aldehyde-fuchsin stain Kit (Solarbio G1593), and the images were obtained and analyzed as described in above section.

### Determination of specific antibodies after rEM-LAP treatment

The specific antibodies were determined by indirect ELISA after treatment with rEM-LAP (19). Blood samples were collected immediately before sacrifice. Serum antibody titers were determined by indirect ELISA against rEM-LAP, and the serum obtained after treatment was diluted 1:4000 before measurement. The specific antibodies were detected with HRP-conjugated goat anti-mouse IgG1, lgG2a, lgG, lgM, lgA, and lgE. TMB was added and incubated at room temperature for 10 min (16). The absorbance at 450 nm was measured by a microplate reader (TECAN, Switzerland).

### Determinations of cytokine production

Serum interferon gamma (IFN-γ) and interleukin-4 (IL-4) were measured by ELISA according to the manufacturer’s instructions (R&D Systems, Minneapolis, MN, USA). The absorbance at 450 nm was measured by a microplate reader (TECAN, Switzerland).

### Statistics

Statistical analyses were performed using SPSS 22.0 and Microsoft Excel. All measurement data were expressed as mean ± standard deviation (SD). Comparison between two groups was performed using the independent sample T-test; while that among multiple groups was performed using the one-way analysis of variance (ANOVA), and the homogeneity of variance was tested by LSD. *P <*0.05 was considered statistically significant.

## Results

**Figure 1:**
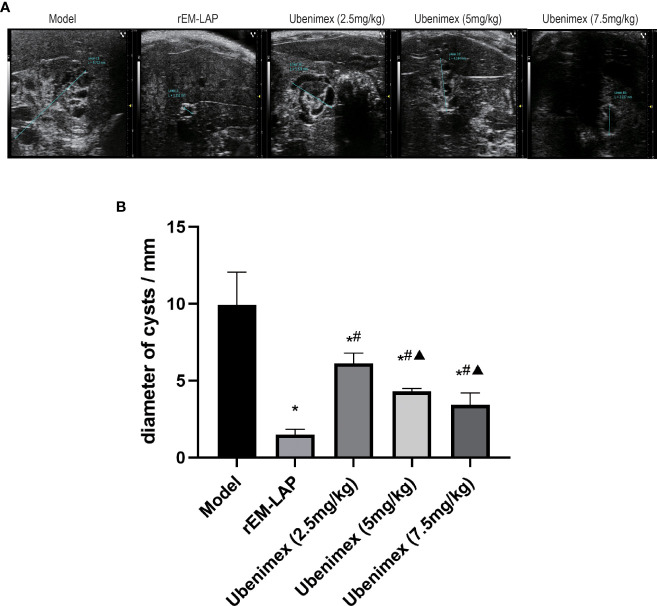
Ultrasonography. **(A)** Cysts in different mouse models by Ultrasonography. **(B)** The maximum diameter was measured by Ultrasonography. (*- p<0.05 v.s. Model group; ^#^- p<0.05 v.s. rEM-LAP group; ^▲^: p<0.05 v.s. 2.5 mg/kg Ubenimex group. n=6, α=0.05).

In this study, the therapeutic effect of rEM-LAP in a mouse model infected with *E.* multilocularis was assessed and verified using Ubenimex, a broad-spectrum inhibitor of LAP. LAP is an aminopeptidase expressed in a variety of species, and the amino acid sequence and spatial structure differ substantially between humans, mice and *E. multilocularis* protoscoleces (20). The bioinformatics sequence alignment results also indicate that EM-LAP is different from host-LAP (**Figure 1S**). Thus, the vaccine is unable to recognize the autologous antigen and the specificity and safety are ensured.

### The therapeutic effect of rEM-LAP

The ultrasonic results show that liver cysts develop and invade into surrounding tissues in the mouse model (**Figure 1A**). Many cysts of different sizes are observed in the model group, most of which are present in the liver. Compared to the model group, the maximum diameter of cysts is significantly decreased in the rEM-LAP group. The same phenomenon is also observed in the Ubenimex group, and the maximum diameter decreases in a dose-dependent manner (**Figure 1B**). It is clear that the therapeutic effect of Ubenimex is not as good as that of rEM-LAP (**Figure 1B**).

**Figure 2:**
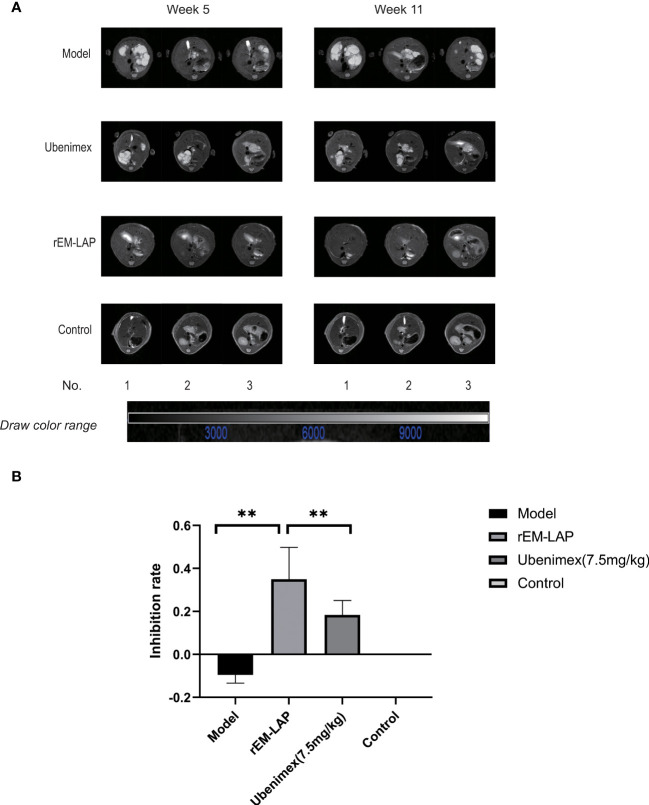
Both rEM-LAP and Ubenimex inhibited cyst growth in livers of mice infected with *E. multilocularis* protoscoleces. **(A)** MRI of the abdomens of mice treated with rEM-LAP IOCV and Ubenimex (7.5 mg/kg) I.P for 6-10 weeks as described in Materials and Methods. **(B)** The cyst growth inhibition rate (IR%) calculated from MRI results. **p<0.01.

The MRI results show that cysts are distributed throughout the liver within 5 weeks in all groups, but the size differs substantially. At week 11, the growth of cysts is significantly inhibited in both rEM-LAP and Ubenimex groups compared to the model group, and it is more pronounced for mice treated with rEM-LAP (**Figure 2A**). In this study, the growth inhibition rate (IR%) is used to represent the therapeutic effect. As expected, the IR% value of the rEM-LAP group (34.95%) is much higher than that of the Ubenimex group (18.35%), and a negative IR% (-9.5%) is observed in the model group (**Figure 2B**).

**Figure 3:**
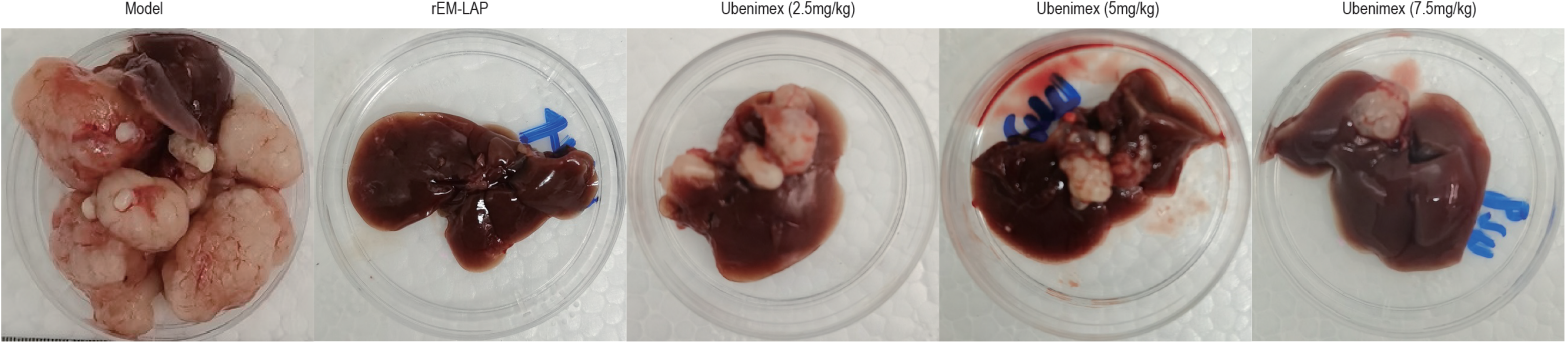
Both rEM-LAP and Ubenimex inhibited cyst growth in the livers of mice infected with E. multilocularis protoscoleces in the late AE stage. Lives excised at week 17 from mice treated with PBS; rEM-LAP; 2.5 mg/kg Ubenimex; 5 mg/kg Ubenimex; and 7.5 mg/kg Ubenimex.

Mice treated with rEM-LAP and Ubenimex were sacrificed at week 17 (60 days) and their livers were excised and photographed. A large number of cysts of different sizes are observed in the model group (**Figure 3**). However, the number and size of cysts are decreased in most livers of mice treated with rEM-LAP and Ubenimex, and it is even absent in some livers. Thus, treatment with rEM-LAP and Ubenimex leads to a substantial decrease in the size and number of cysts in the liver, especially in the rEM-LAP group.

**Figure 4:**
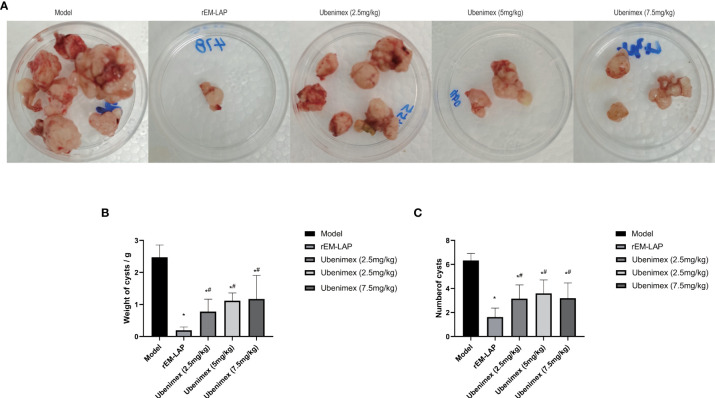
Therapeutic effect of rEM-LAP. **(A)** The size and number of cysts at week 17. **(B)** The weight of cysts in different groups. **(C)** The number of cysts in different groups. (* p<0.05 v.s. Model group; #: vs p<0.05 v.s. rEM-LAP group. n=6, α=0.05).

There is a significant difference in the cysts from the abdomen and liver of mice treated with rEM-LAP, Ubenimex and PBS (**Figure 4A**). Compared to the model group, the weight (**Figure 4B**) and number (**Figure 4C**) of cysts are significantly decreased in both rEM-LAP and Ubenimex groups, and a more pronounced inhibiting effect is observed in the rEM-LAP group. However, it is important to note that the dose of Ubenimex has no significant effect on the weight and number of cysts. These results suggest that rEM-LAP may be a viable treatment option for the infection of *E. multilocularis* protoscoleces.

**Figure 5:**
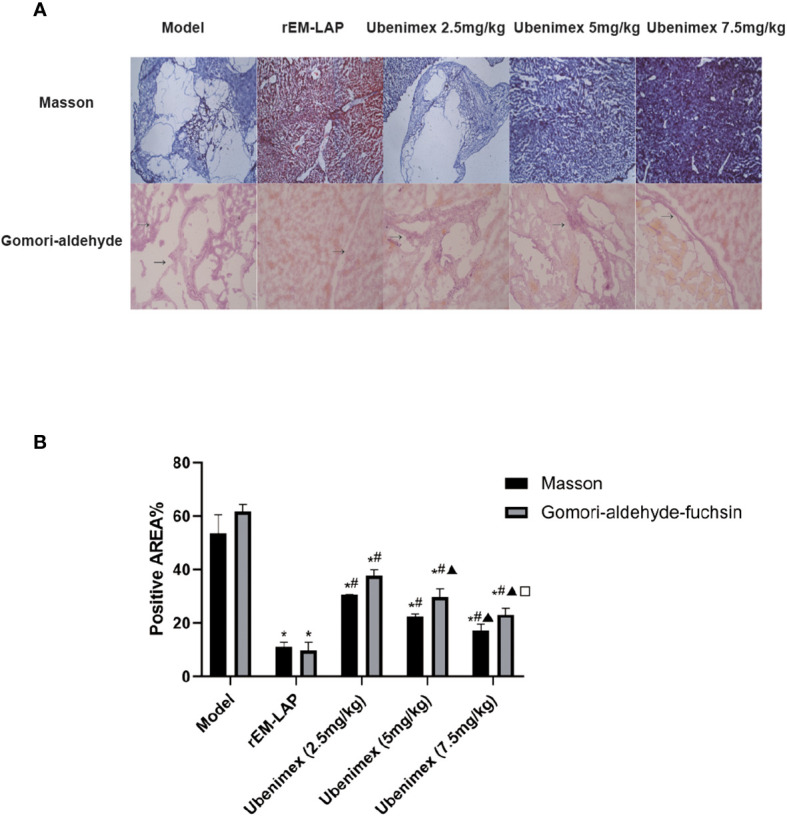
Treatment with rEM-LAP or Ubenimex inhibited the infiltration and fibrosis of cysts in livers of mice infected with E. multilocularis protoscoleces. **(A)** Representative Masson–stained and Gomori-aldehyde-fuchsin–stained cyst sections from mice treated with rEM-LAP, Ubenimex, and PBS (200×) **(B)** Positive staining area (%) is presented in each image. The results are obtained from several images of 3 mice for each specific staining (* p<0.05 v.s. Model group; ^#^ p<0.05 v.s. LAP group; ^▲^ p<0.05 v.s. 5 mg/kg Ubenimex group; □ p<0.05 v.s. 2.5 mg/kg Ubenimex group. n=3, α=0.05).

Treatment with rEM-LAP can inhibit the growth and invasion of cysts in mice infected with *E. multilocularis* protoscoleces, as evidenced by the decreased levels of collagenous and elastic fibers (**Figure 5A**). The levels of Masson–stained and Gomori-aldehyde-fuchsin–stained cysts are decreased by about 42% and 52% in livers of rEM-LAP treated mice, respectively. Thus, the levels of fibrosis and infiltration in livers of mice treated with rEM-LAP and Ubenimex are dramatically decreased compared to untreated mice (**Figure 5B**).

### Immunological characteristics of rEM-LAP proteins

**Figure 6:**
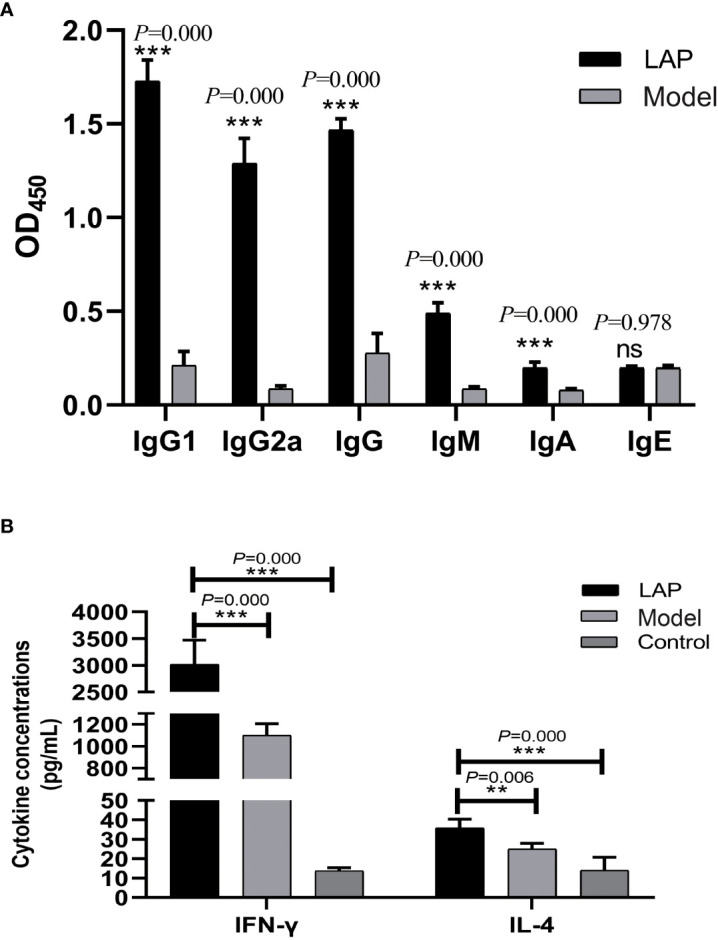
Immunological characteristics determined by ELISA. **(A)** The levels of antibodies. **(B)** Serum cytokine concentrations. **p<0.01, ***p<0.001, ns, no statistical significance.

Compared to the model group, treatment with rEM-LAP leads to a significant increase in the levels of specific IgG1 (*t*=35.78, *p*=0.000) and IgG2a (*t*=17.59, *p*=0.000) against LAP antigen (**Figure 6A**). The indirect ELISA shows a significant increases in the levels of serum anti-LAP IgG (*t*=18.546, *p*=0.000), IgM (*t*=23.556, *p*=0.000) and IgA (*t*=15.387, *p*=0.000) antibodies. However, there is no significant difference in the levels of anti-LAP IgE antibody (*t*=-0.256, *p*=0.978).

### Secretion of serum cytokines

The serum levels of IFN-γ and IL-4 were tested by ELISA. To eliminate the effect of Freund’s adjuvant, the normal control group (NC) was included in the comparison. Compared with model and NC groups, the concentrations of IFN-γ cytokine (*p*=0.000 v.s. model group; *p*=0.000 v.s. NC group; *F*=945.582) and IL-4 (*p*=0.006 v.s. model group; *p*=0.000 v.s. NC group; F=567.828) are significantly increased in mice treated with rEM-LAP (**Figure 6B**).

## Discussion

AE is one of the most prevalent parasitic diseases in the world and it develops like a tumor and invades adjoining tissues of the host. Vaccination is an effective broad-spectrum treatment of AE as it can not only induce specific immunity response but can also inhibit the growth and metabolism of cysts (21). However, it remains challenging to treat *E. multilocularis* infection and new targets and therapeutic approaches are required to inhibit the infiltration and fibrosis of cysts in the liver. The results of this study have demonstrated that rEM-LAP can be an effective therapeutic vaccine for AE in mice infected with *E. multilocularis.*

LAP is an important protease present in many species and participates in leucine degradation as a key enzyme. It is involved not only in proteolysis but also in macromolecular assembly and colonization (22). There is evidence that the LAP of *malaria parasite* is a potential therapeutic target because of its important role in ontogeny and pathopoiesia (23) and it is also an important vaccine epitope to protect *Fasciola hepatica* (13). Our previous study has revealed that EM-LAP is related to the proteolysis and growth and it is the dominant epitope that could induce immune response and is involved in the invasion and fibrosis (Wang et al., 2021). EM-LAP belongs to the M17 family and can be inhibited by EDTA and Ubenimex. Thus, EM-LAP plays an important role in the growth, development and invasion of *E. multilocularis* in the liver.

In order to verify the therapeutic effect of rEm-LAP and whether inhibition of EM-LAP could reduce the growth, invasion and fibrosis of cysts, specific antibodies are induced and their effects are evaluated. It is found that rEM-LAP has great potential to reduce the infection *of E. multilocularis* by inhibiting the growth, invasion and fibrosis of cysts in the liver. Suppression of EM-LAP activity by rEM-LAP provides an effective strategy to protect against *E. multilocularis* infection and cyst growth (**Figures 1A, B**, and **2**). Cytokines are released and the metabolism is inhibited by activating specific antibodies. rEM-LAP could inhibit cyst growth and invasion into adjoining tissues (**Figures 1A, B**, and **2**). Intravenous injection of rEM-LAP can inhibit cyst growth and even eliminate the cysts in mice infected with *E. multilocularis* protoscoleces (**Figures 2**, **3B**, and **4**). Treatment with Ubenimex leads to the elimination of cysts or the reduction in the number and size of cysts (**Figure 3**; **4**). The therapeutic effect of rEM-LAP may result from the specific immunity response to induce cytokines and alter the immune microenvironment such as Th1-type and Th2-type immune response balance. Mice treated with rEM-LAP also show lower levels of invasion and fibrosis (**Figure 5**). These effects of rEM-LAP on cyst growth can be attributed to its effect on the metabolic disturbance and specific immunity.

The number and size of cysts from the abdomen and liver are perhaps the most intuitive indicators for evaluating the therapeutic and protective effects of rEM-LAP in mice infected with *E. multilocularis protoscoleces*. In most clinical cases, parasite infiltration and growth in liver are evaluated using medical images. However, liver function and the expression of key proteins of *E. multilocularis* are not considered in previous mice models and clinical cases. Liver function is not a particularly sensitive evaluation index in clinical cases and it is only used to determine the preoperative nutritional status of patients with hepatic alveolar echinococcosis and AE-related autologous liver transplantation in order to evaluate the effect of surgery (24, 25). The expression of key proteins of *E. multilocularis* is not measured in animal model (7, 16, 21), because few substances are secreted into the tissue due to the presence of insect protein and cyst and no specific antibody is currently available for verification. For these reasons, we have evaluated not only the sizes of cysts but also parasite infiltration and growth in the liver through MRI, ultrasound and pathological examination.

In this study, a new vaccine rEM-LAP is proposed for the treatment of AE in mice infected with *E. multilocularis* protoscoleces, and the therapeutic effect is verified with Ubenimex. The results show that rEM-LAP inhibits the activity of LAP protease to reduce *E. multilocularis* protoscoleces growth and cyst invasion and induces strong specific immune response (**Figures 3B**, **4**–**6**). Thus, EM-LPA is closely associated with the infiltration and fibrosis in the host liver, but the mechanism is still unclear yet.

The results also show that treatment with rEM-LAP is effective in all experimental groups because it can not only inhibit LAP activity to reduce cyst growth and invasion, but it can also induce strong and long-lasting specific immunity response. **Figure 6** reveals that treatment with rEM-LAP induces significant serum antibodies (IgG1, IgG2a, IgG, IgM and IgA). IFN-γ is secreted primarily by Th1 lymphocytes, while IL-4 is secreted primarily by Th2 lymphocytes. In this study, treatment with rEM-LAP leads to a Th1 and Th2 mixed-type immune response, which contributes to substantially reducing the growth of cysts and the parasite load in the liver of *E. multilocularis* infected mice, and it can also maintain the immune response balance without activating cytotoxic T cells (26). Previous studies have suggested that abundant IgE is activated in the infection with parasite (27), the serum level of IgE is low in this study, which is probably because treatment with rEM-LAP mainly mediates IgG dominated immunoreaction and maintains a Th1 and Th2 mixed-type immune response.

Ubenimex is a broad-spectrum LPA protein inhibitor which can restrain the LAP proteins in many species, such as *Leishmania*, human liver *fluke*, *plasmodium* and *E. multilocularis*, and inhibit the migration and invasion in gastric cancer by alleviating the activity of the CD13/NAB1/MAPK pathway (28). It can also inhibit cell proliferation, migration and invasion in prostate cancer, human cervical cancer and non-small lung cancer by inhibiting the expression of APN and inducing autophagic cell death (29–31). Thus, LAP may be related to the invasion and infiltration, and *E. multilocularis* infection and cyst growth are similar to above cancers, implying that they may have common mechanisms and pathways.

Masson staining and Gomori-aldehyde-fuchsin staining were performed to evaluate the invasion and fibrosis levels of the host liver. LAP as an important peptidase can synthesize the proteins involved in the infiltration and fibrosis from the amino acids of the host, and thus inhibition of the synthesis of these amino acids can prevent the infiltration and fibrosis. Some other studies have reported that collagenous and elastic fibers are positively correlated in some invasive diseases, such as malignant tumor and hepatic fibrosis-renal tubular ectasia syndrome (32, 33). LAP can catalyze the hydrolysis of leucine residues from the amino termini of protein or peptide substrates and then produce the tumor or cyst protein (34, 35). LAP3 is involved in the progression and metastasis of breast cancer by upregulating the expression of fascin and matrix metalloproteinases-2/9 (MMP-2/9) (34). Fascin is an actin cytoskeletal protein that can promote actin filament from filopodial and invadopodial protrusions, which are the major components of cell synapses. Cell synapse plays an important role in the perception of the external environment and cell migration and fibrosis (36, 37). MMP-2/9 is a member of the MMPs family that is highly expressed in colorectal cancer, gastric cancer, and nasopharyngeal carcinoma and closely related to tumor invasion and fibrosis, and knockdown of LAP3 can reduce MMP-2/9 expression and consequently cell invasion (38). Similarly, inhibition of leucine aminopeptidase suppresses the invasion and fibrosis of ovarian cancer by down-regulating the expression of fascin and MMP-2/9 (35). In this study, we have found that *E. multilocularis* protoscoleces grow like a malignant tumor in the host and inhibition of EM-LAP may reduce cyst invasion and fibrosis by reducing the synthesis of key amino acids such as fascin and promoting the synthesis of collagenous and elastic fibers (21, 34, 39). Our future study will focus on the mechanism of how EM-LAP reduces cyst invasion and fibrosis.

LAP is one member of the M17 peptidase family and it could act as an ideal target antigen in the diagnosis and prevention of parasitic diseases (e.g., malaria and fascioliasis) because of its good immunogenicity. However, little is known about its role in *E. multilocularis*. Our previous study has suggested that Em-LAP could be a potential vaccine antigen of *E. multilocularis* and a protective antigen to reduce cysts (21). Some studies have also reported that LAP is related to migration, tissue invasion, and nutrition intake in some parasitic diseases (12, 40, 41). The high expression of LAP is related to cell proliferation, migration, and invasion in some cancers (42–44), and *E. multilocularis* protoscoleces grow like a malignant cancer in the host liver. Therefore, it is also necessary to clarify whether EM-LAP inhibitor is effective in invasion and fibrosis of cysts in the host liver. In this study we evaluated parasite infiltration and growth in the host liver using MRI, ultrasound and pathological examination. The results show that treatment with rEM-LAP can reduce not only cyst growth and metabolism in the host liver, but also pathology-associated infiltration process and thus the damage, invasion, and fibrosis in the liver of mice infected with *E. multilocularis*. LAP is an aminopeptidase found in many species, but it is highly species specific in different species such as *E. multilocularis*, human and mouse. Thus, EM-LAP as a therapeutic vaccine shows specific immunity and the therapeutic efficiency is better than LAP broad spectrum inhibitor treatment. More recent studies have focused simply on the parasiticidal effect (15, 45) and evaluated only the *protoscoleces* number and cyst size *in vivo* or *in vitro* without considering parasite invasion, migration and fibrosis in the host (46, 47). This study measured cyst size and invasion level in the host to evaluate the therapeutic efficiency of EM-LAP.

## Conclusions

In conclusion, LAP plays a role in the invasion and fibrosis of cysts, and treatment with rEM-LAP can regulate the metabolism of *E. multilocularis* and induce specific immune response as an antigenic epitope. Thus, rEM-LAP is a potential candidate for the treatment for AE.

## Data availability statement

The raw data supporting the conclusions of this article will be made available by the authors, without undue reservation.

## Ethics statement

All animal experiments were performed in compliance with the regulations of the Ministry of Science and Technology of China and approved by the Experimental Committee of Qinghai University (QHDX-2019-09).

## Author contributions

ZZ: writing - original draft, writing - review & editing, and visualization. PZ: writing - original draft. YM: investigation and supervision. LW: investigation and supervision. ZC: investigation and supervision. SD: investigation and supervision. HB: investigation and supervision. BY: investigation. MX: investigation. RL: writing - review & editing and methodology. R-LG: conceptualization and project administration. FT: writing - review & editing and methodology. All authors contributed to the article and approved the submitted version.

## Funding

This work was supported by National Natural Science Foundation of China (81860299), Qinghai Department of Science and Technology (2020-ZJ-963Q), Young and Middle-aged Scientific Research Fund Team Project of Qinghai University School of Medicine (2019-KT-01), and the “Thousand Talents Program” for High-end Innovation of Qinghai Province.

## Acknowledgments

We acknowledge the Research Center for High Altitude Medicine of Qinghai University for providing the experimental platform.

## Conflict of interest

The authors declare that the research was conducted in the absence of any commercial or financial relationships that could be construed as a potential conflict of interest.

## Publisher’s note

All claims expressed in this article are solely those of the authors and do not necessarily represent those of their affiliated organizations, or those of the publisher, the editors and the reviewers. Any product that may be evaluated in this article, or claim that may be made by its manufacturer, is not guaranteed or endorsed by the publisher.
