# Supplementary figures and images for: Therapeutic effect on Alveolar echinococcosis by targeting EM-Leucine aminopeptidase
Source: Front Immunol. 2022 Oct 14;13:1027500. doi: 10.3389/fimmu.2022.1027500 (PMC9614657; doi:10.3389/fimmu.2022.1027500)

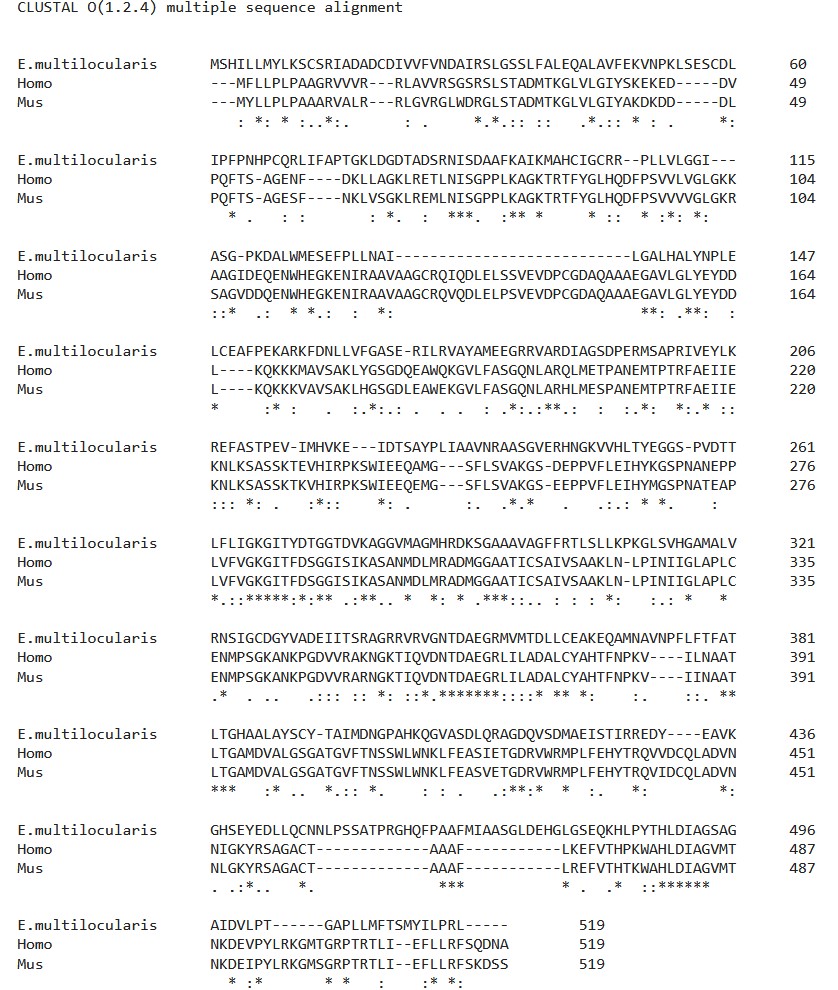

Supplement: Supplementary file 1 [file Image_1.jpeg]

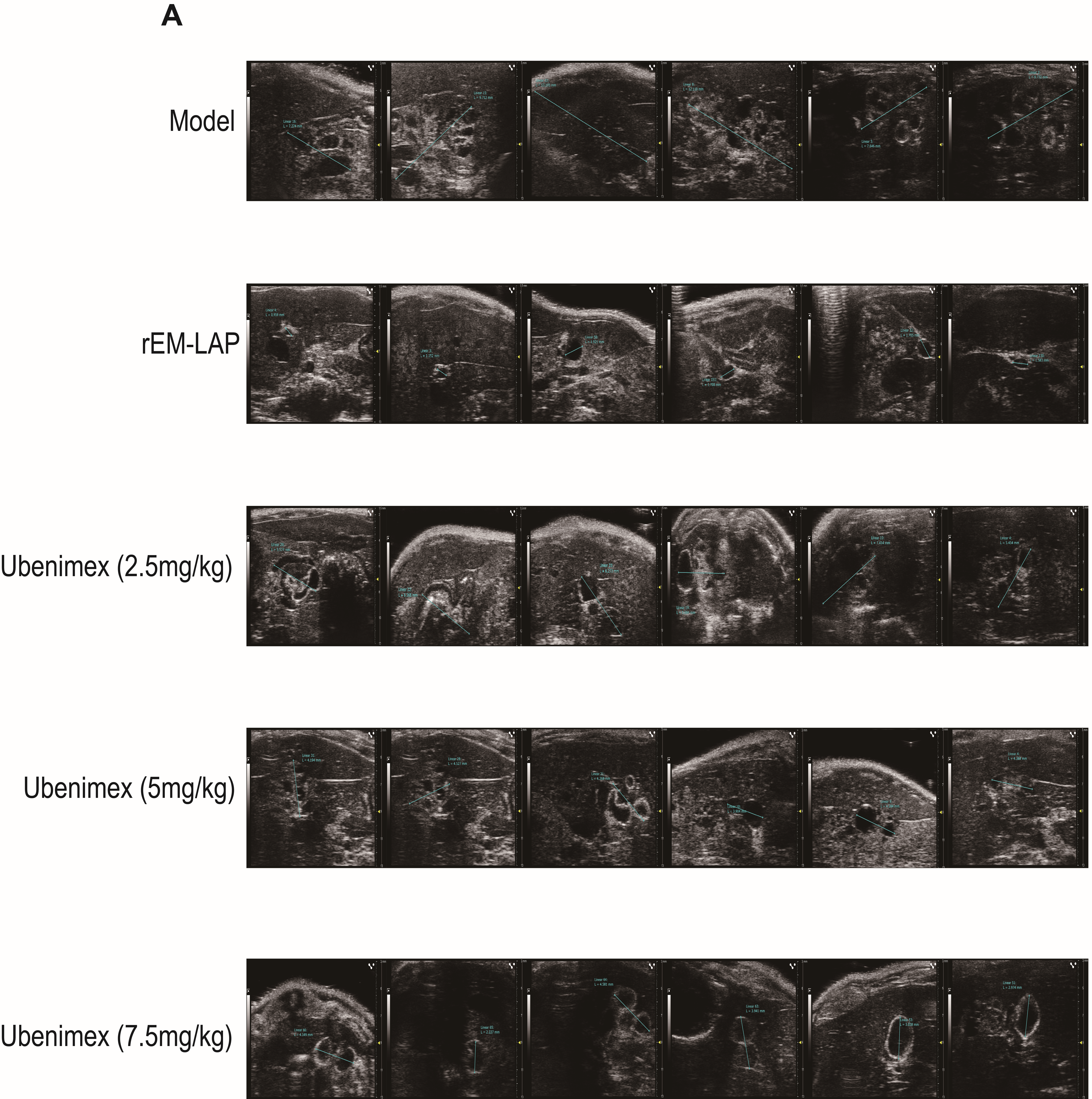

Supplement: Supplementary file 2 [file Image_2.tif]

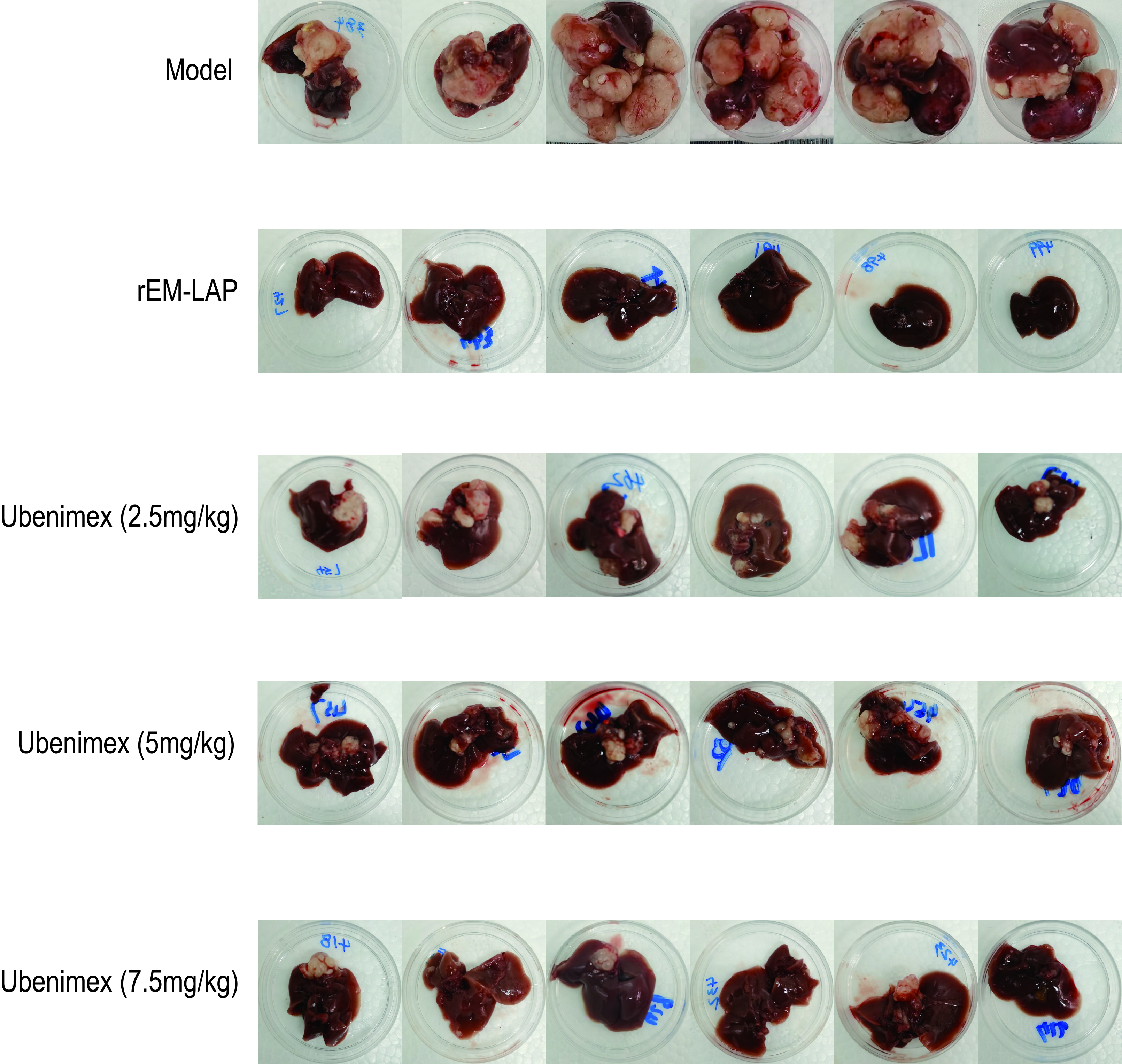

Supplement: Supplementary file 3 [file Image_3.tif]

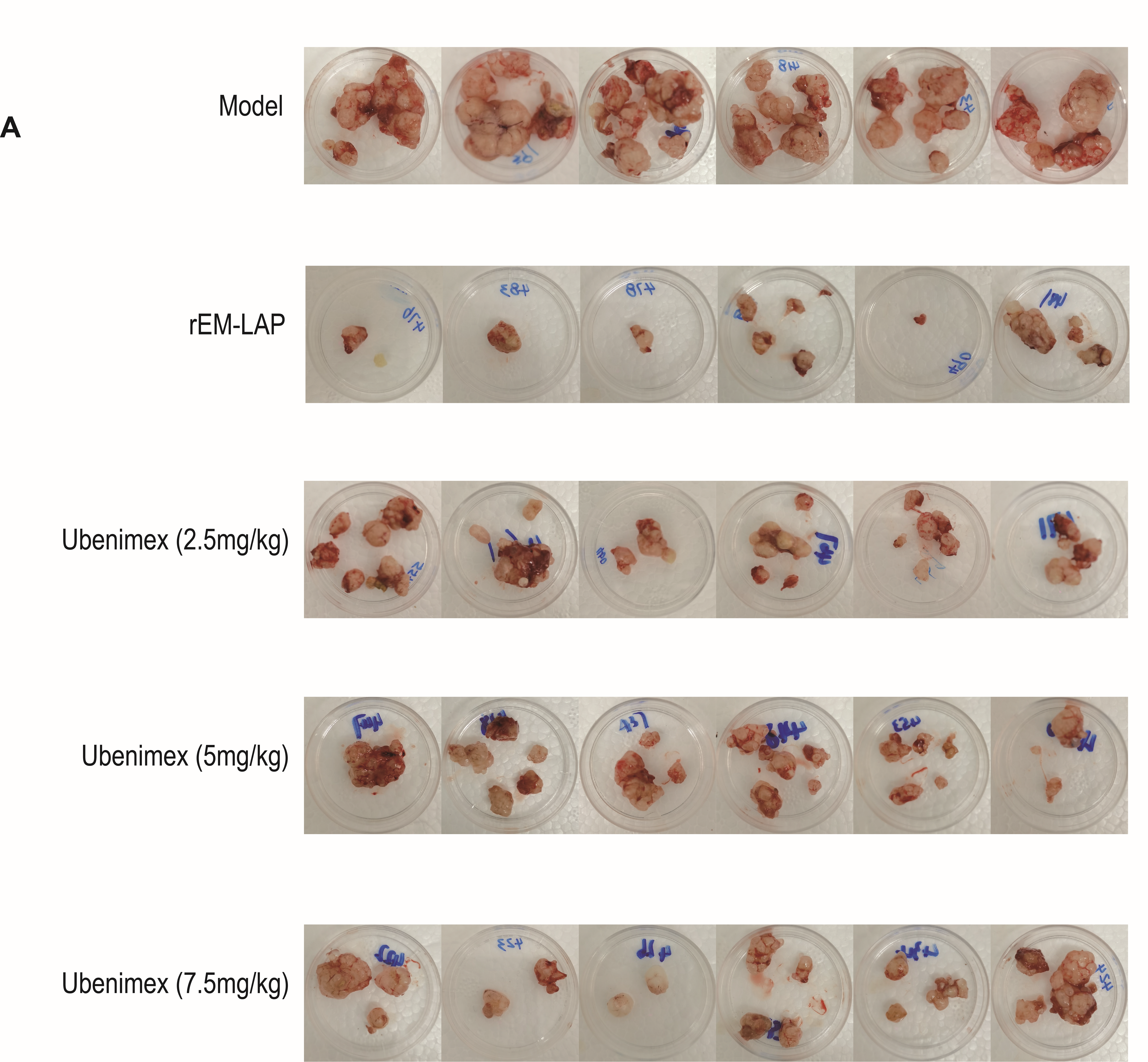

Supplement: Supplementary file 4 [file Image_4.tif]
